# Controlled Copolymerization of Ethylene and Biosourced Comonomers Using Dibenzobarrelene-Based *α*-Diimine Nickel Catalyst

**DOI:** 10.3390/molecules30112402

**Published:** 2025-05-30

**Authors:** Handou Zheng, Junsong Wang, Zonglin Qiu, Chunyu Feng, Haotian Zhou, Guangshui Tu, Haiyang Gao

**Affiliations:** School of Materials Science and Engineering, PCFM Lab, Sun Yat-sen University, Guangzhou 510275, China; zhenghdou@mail.sysu.edu.cn (H.Z.); wangjs33@mail2.sysu.edu.cn (J.W.); qiuzlin3@mail2.sysu.edu.cn (Z.Q.); fengchy28@mail2.sysu.edu.cn (C.F.); zhouht23@mail2.sysu.edu.cn (H.Z.); tugsh@mail2.sysu.edu.cn (G.T.)

**Keywords:** *α*-diimine nickel, copolymerization, biosourced monomers, functional polyethylene

## Abstract

**The** development of earth-abundant nickel-based catalysts is currently one of the greatest challenges for the straightforward synthesis of functionalized polyolefins. With environmental protection concerns, controllable copolymerizations of ethylene with biosourced comonomers derived from castor oil, such as methyl 10-undecenoate (U-COOMe), 10-undecen-1-ol (U-OH), or 10-undecenyl bromide (U-Br), were realized using *α*-diimine nickel catalyst (**Ni-DBB**) with dibenzobarrelene backbone. Catalyst **Ni-DBB** was highly tolerant toward polar comonomers, and functional polyethylenes were successfully prepared. The influences of the polar group, temperature, and comonomer concentration were studied in detail. Catalyst **Ni-DBB** was able to catalyze the copolymerization of ethylene with U-OH to afford high-molecular-weight (~180 kg/mol) functional polyethylene in a controlled fashion. NMR analysis showed that the produced functional polyethylenes were highly branched and had broad melting peaks ranging from 0 to 30 °C. Water contact angle (WCA) measurements showed that the surface of the obtained hydroxyl-functionalized polyethylene changed from hydrophobic to hydrophilic with the introduction of the comonomer U-OH.

## 1. Introduction

Polyolefins, especially polyethylene (PE), are commercially the most important family of polymeric materials because of their excellent mechanical, physical, and chemical properties [1]. However, the full non-polar nature of the polyolefins also limits their wider applications. Compared to their non-functionalized analogues, functional polyethylenes exhibit beneficial properties such as adhesion, printability, miscibility, and toughness [2,3]. There are three major approaches for the synthesis of functional polyolefins, including free radical copolymerization of ethylene and polar monomers under harsh conditions [4,5], post-functional modification of polyolefins [6], and direct copolymerization of ethylene and polar monomers through coordination polymerization under mild conditions [7]. Undoubtedly, the direct copolymerization with polar monomers is the most promising approach, which allows the preparation of functional polyolefins with controllable microstructure [8].

Transition metal catalysts play a crucial role in the coordination copolymerization of ethylene with polar comonomers. However, early transition metals, such as those in groups 4–6, are often inactive for the direct copolymerization of ethylene and polar comonomers due to the high oxophilicity of their metal centers [9,10]. In contrast, late transition metal catalysts are ideal candidates for the copolymerization of ethylene and polar monomers because of their low oxophilicity [11,12]. Several late transition metal Ni/Pd catalysts, such as *α*-diimine [13,14,15], phosphine-sulfonate [16,17,18,19], salicylaldiminate [20,21,22,23,24], and phosphino-phenolate [25,26,27,28,29], have been developed for effectively copolymerizing ethylene and polar comonomers. A notable example is the *α*-diimine nickel and palladium catalysts (also known as Brookhart-type catalysts), which often produce branched or highly branched copolymers containing a portion of, or all, terminal polar groups due to chain walking [30,31]. Generally, the *α*-diimine nickel catalysts are more active for ethylene homopolymerization and have lower chain walking ability than palladium analogues. However, nickel catalysts have lower tolerance toward polar comonomers than palladium catalysts [13,32]. Therefore, the copolymerization of ethylene and polar comonomers using *α*-diimine nickel catalysts remains a significant challenge due to their relatively high metal oxophilicity. This challenge can be addressed by employing: (a) comonomers bearing sterically hindered functional groups or functional groups positioned far from the polymerizable double bond through long spacers [33,34]; (b) comonomers with protected functional groups [35,36]; or (c) catalytic systems with enhanced tolerance toward polar groups [37,38].

Few *α*-diimine nickel catalysts have been developed to directly copolymerize ethylene and polar comonomers, although Ni-based catalysts are low-cost and abundant in the Earth’s crust [11,39]. Increasing bulky steric hindrance around the metal center is an effective approach to enhance the tolerance of the nickel metal center toward polar groups [40,41]. Introducing bulky anilines such as 2,6-dibenzhydrylanilines and 8-arylnaphthylamine has been widely developed in the design of *α*-diimine nickel catalysts for copolymerization of ethylene and polar monomers [42,43,44]. Besides, installing bulky backbones such as camphyl [45,46] and dibenzo-/dinaphthobarrelene backbones [47,48,49,50] has also been developed by our group for the copolymerization of ethylene and polar monomers.

With carbon peaking and carbon neutrality goals, as well as environmental protection concerns, the use of biosourced monomers in polymer synthesis is receiving increasing attention [51,52,53]. Biosourced monomers, such as 10-undecenoic acid (U-COOH), derived from the pyrolysis and extraction of castor biomass oil, have attracted considerable interest in the synthesis of functional polymers because of their biosourced character [54]. It has served as a key precursor in the industrial production of pharmaceuticals, consumer goods, and specialty polymers [55,56]. The flexible long-chain alkyl moiety in its molecular structure contributes to improved flexibility of the resultant polymeric materials. Furthermore, a series of 10-undecenoic acid derivatives can be obtained through chemical transformations such as esterification or other derivatization reactions, which function as critical intermediates in developing biodegradable elastomers, hydrogels, and environmentally benign adhesives [57,58].

**Scheme 1:**
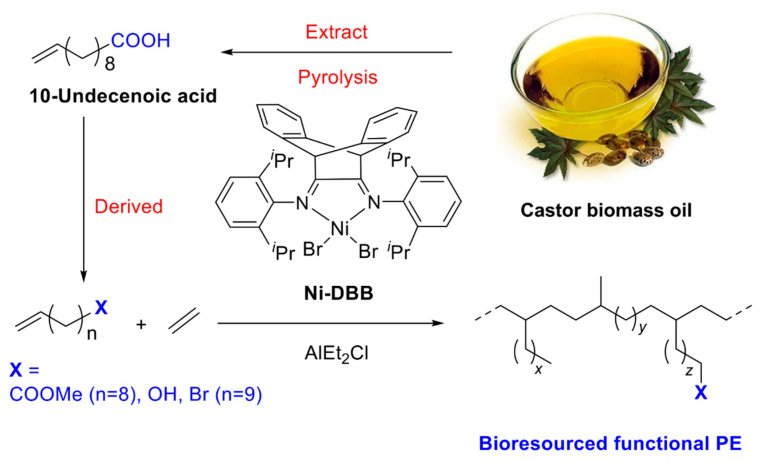
Copolymerization of ethylene and biosourced comonomers derived from castor oil using dibenzobarrelene *α*-diimine nickel catalyst.

In this contribution, a dibenzobarrelene *α*-diimine nickel catalyst was used in the copolymerization of ethylene and biosourced 10-undecenoic acid derivatives (CH₂=CH–(CH₂)ₙ–X, X = COOMe (n = 8), OH, and Br (n = 9)) (Scheme 1). Bulky dibenzobarrelene backbone of the ligand effectively enhanced the tolerance of the nickel catalyst towards polar comonomers, and functional polyethylenes were successfully prepared. More importantly, hydroxyl-functionalized polyethylene with high molecular weight (*M*_n_~180 kg/mol) was synthesized precisely in a controlled fashion.

## 2. Results and Discussion

### 2.1. Copolymerization of Ethylene and Comonomers

Our group has previously developed dibenzobarrelene-based *α*-diimine nickel and palladium catalysts. Introduction of bulky dibenzobarrelene backbone can enhance thermal stability and controlled fashion in *α*-diimine nickel-catalyzed ethylene polymerization [47]. Besides, the dibenzobarrelene-based *α*-diimine palladium catalyst can catalytically prepare functional polyolefins by controlled copolymerization of ethylene and a variety of acrylate comonomers [48,49,51]. Prior to this work, we attempted the copolymerization of ethylene with 10-undecylenic acid (U-COOH). However, only trace amounts of polymer were obtained (entry 13 in Table 1). Herein, the copolymerizations of ethylene with biosourced 10-undecenoic acid derivatives (CH_2_=CH–(CH_2_)_n_–X, X = COOMe (U-COOMe, n = 8), OH, and Br (U-OH or U-Br, n = 9)) using dibenzobarrelene-based *α*-diimine nickel catalyst **Ni-DBB** were carried out. In these copolymerization systems, a large amount of AlEt_2_Cl (Al/Ni = 1600) as a cocatalyst was used compared to ethylene homopolymerization (Al/Ni = 600) [47], because a large amount of alkyl aluminum reagents is helpful for protecting the polar groups for successful copolymerization. [59,60,61,62,63]. These functional groups in the olefins preferentially coordinate to Al, forming stable and protected aluminates that serve as true comonomers for successful copolymerization, and deprotection to the free functionality is accomplished by acid wash upon termination of polymerization [64,65].

**Figure 1:**
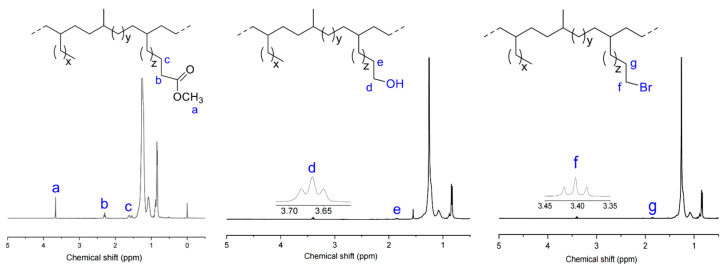
^1^H NMR spectra of the obtained functional polyethylenes in CDCl_3_ (entries 3, 7, and 11 in Table 1).

As shown in Table 1, copolymerizations of ethylene and three biosourced comonomers derived from castor oil with different polar groups were conducted in a temperature range from 20 to 65 °C. Excluding the amount of AlEt_2_Cl used as the activator for ethylene polymerization (Al/Ni = 600, 1.2 mmol), an additional 2.0 mmol of AlEt_2_Cl was added in the copolymerization system, resulting in an [Al]/[polar monomer] ratio of 1. The polar comonomer incorporation into the copolymer was identified by ^1^H NMR spectroscopy, and no homopolymer of polar comonomer was present in the copolymerization products. For the ethylene/U-OH copolymer, the signal corresponding to the hydroxyl protons is difficult to observe. Therefore, the incorporation (mol%) of hydroxyl-containing monomers in the copolymer is typically calculated by integrating the methylene proton signals (–C***H***_2_OH) relative to the signals of other protons in the ^1^H NMR spectrum (see Section 3.3) [39,66,67]. As shown in Figure 1, ^1^H NMR spectra of the obtained functional polyethylene clearly show characteristic peaks for U-COOMe (***H***_a_: 3.60 ppm; ***H***_b_: 2.30 ppm; ***H***_c_: 1.61 ppm), U-OH (***H***_d_: 3.66 ppm; ***H***_e_: 1.85 ppm), and U-Br (***H***_f_: 3.40 ppm; ***H***_g_: 1.85 ppm), respectively, which proves the successful copolymerization of ethylene with U-COOMe, U-OH, or U-Br [47,49,68].

**Figure 2:**
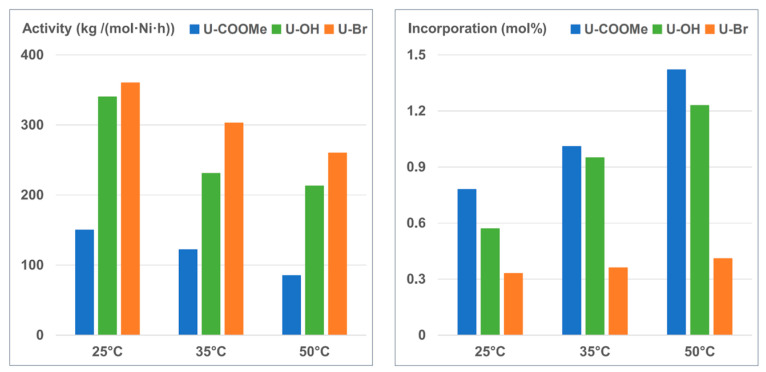
The effects of polar groups on copolymerization activity and comonomer incorporation at different temperatures.

The effects of polar groups on copolymerization were first examined. As shown in Table 1 and Figure 2, copolymerization activity increased but incorporation of polar comonomers decreased in the order of U-COOMe > U-OH > U-Br under identical reaction conditions, strongly indicating that the ester group -COOMe exhibited the strongest poisoning effect on the nickel metal center. This was attributed to the easiest *σ*-interaction between the ester group and the nickel metal center [32,69,70]. Additionally, copolymer molecular weight followed the order of U-OH > U-COOMe > U-Br. Although copolymerization of ethylene and U-Br showed the highest activity, the resultant copolymer molecular weight was the lowest. This is attributed to the accelerated chain transfer reaction caused by the electron-withdrawing Br group [71,72].

The influences of reaction parameters on the copolymerization of ethylene and polar comonomers using the catalyst **Ni-DBB** were further investigated. As shown in Table 1, the copolymerization activity and the molecular weight of copolymers decreased, but the incorporation of the polar monomer increased with an increase in the temperature from 20 to 65 °C. Besides, the copolymers showed relatively narrow distribution (PDI < 1.65), indicating a controlled copolymerization fashion [73]. The molecular weight distribution of the copolymer also became broader with increasing temperatures. These observations suggested that elevated temperature was favorable for the incorporation of polar comonomers and the acceleration of chain transfer. To further illustrate the polar group tolerance of the dibenzobarrelene *α*-diimine nickel catalyst, copolymerizations were further conducted at various polar monomer concentrations using the catalyst **Ni-DBB** (Table 2). Increasing the comonomer concentration resulted in a lower [Al]/[polar monomer] ratio (0.3~0.5), which led to an increase in the incorporation of comonomers, whereas the copolymerization activity and the molecular weight of copolymers decreased (entries 3, 6, 8, and 9 in Table 2). Besides, copolymerization of U-Br was conducted at a higher comonomer concentration. Lowering the relative additional Et_2_AlCl/comonomer ratio to 0.3 still allowed the formation of a large amount of copolymer (entry 9 in Table 2), which was a result of reducing the poisoning effect of Br toward the nickel metal center compared to the ester and hydroxyl groups [69,74].

**Figure 3:**
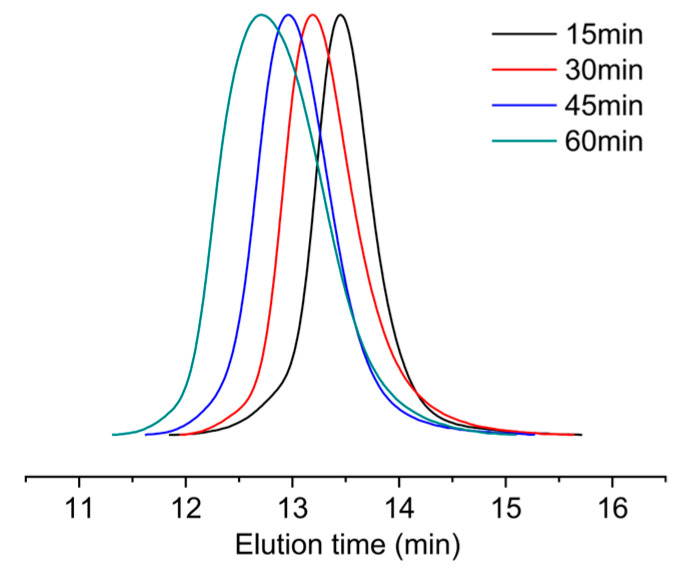
GPC curves of E/U-OH copolymers obtained at different reaction times.

**Figure 4:**
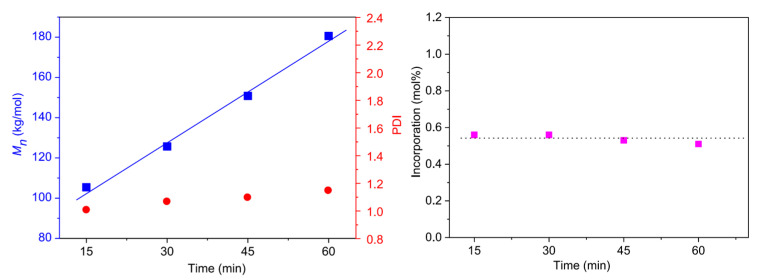
Plots of *M*_n_, PDI, and incorporation of U-OH as a function of reaction time using **Ni-DBB**/Et_2_AlCl at 20 °C.

The experimental results, as mentioned above, clearly showed that copolymerization of ethylene and U-OH was the most efficient in producing narrowly dispersed copolymers among the three comonomers. The controlled copolymerization process of ethylene and U-OH using the catalyst **Ni-DBB** was further studied at 20 °C. Figure 3 shows symmetric GPC traces of the copolymers produced at different reaction times without tailing peaks, which shift to the higher molecular weight region with prolonged reaction time. Plots of number-average molecular weights (*M*_n_) as a function of reaction time (Figure 4) also show that *M*_n_ grows linearly with the polymerization time, and *M*_w_/*M*_n_ (PDI) values are below 1.20. Further, the incorporation of U-OH into copolymers obtained at different time periods remains nearly constant (∼0.57 mol%), indicating that the comonomer U-OH is uniformly incorporated into polymer chains [48,51]. The hydroxyl functionalized polyethylene with a high molecular weight up to 180 kg/mol can be produced. Therefore, the dibenzobarrelene-based *α*-diimine nickel catalyst (**Ni-DBB**) is able to catalyze the copolymerization of ethylene and U-OH in a controlled fashion.

### 2.2. Microstructures of Functional Polyethylene

**Figure 5:**
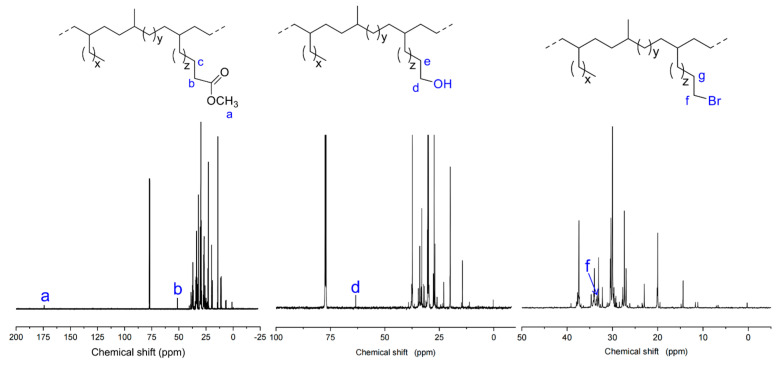
^13^C NMR spectra of the obtained functional polyethylenes in CDCl_3_ (entries 3, 7, and 11 in Table 1).

^1^H NMR spectroscopy analysis (Figure 1) showed that the obtained functional polyethylene was highly branched (75~101/1000 C), which was different from the semi-crystalline polar polyethylene produced by Coates and co-workers [75]. To gain deep insight into the definitive microstructure of the produced functional polyethylene, the functional polyethylenes were analyzed by ^13^C NMR spectroscopy (Figure 5). ^13^C NMR spectra of the obtained functional polyethylene show characteristic peaks for U-COOMe (***C***_a_: 174.35 ppm, ***C***_b_: 51.49 ppm), U-OH (***C***_d_: 63.38 ppm), and U-Br (***C***_f_: 33.53 ppm), respectively, which prove the successful copolymerization of ethylene and U-OH or U-Br. The branching distribution is quantitatively calculated on the basis of previous resonance assignments [76,77]. The obtained copolymers mostly contain methyl branches and long-chain branches, and the total content of other short branches, such as ethyl, propyl, butyl, and amyl, was very low. This is attributed to the chain walking mechanism previously reported in our work [78].

**Figure 6:**
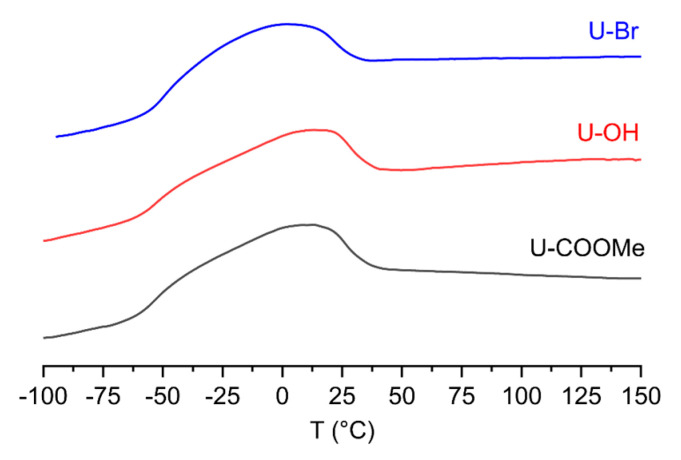
The DSC curves of functional polyethylenes (entries 3, 7, and 11 in Table 1).

Differential scanning calorimetry (DSC) analyses were conducted to examine the thermal behavior of the copolymers. As shown in Figure 6, the DSC curves of these copolymers (up to 150 °C) clearly show that no polyethylene homopolymer is present in the copolymerization products. Moreover, the functional polyethylenes have very broad melting endotherms, which are attributed to inhomogeneous branching distribution and different branching lengths. Generally, three kinds of functional polyethylenes have nearly the same DSC profiles, indicating nearly the same branching structure. This observation also indicates that the polar comonomer does not impact the chain walking process during copolymerization.

### 2.3. Hydrophilic Property of Functional Polyethylene

**Figure 7:**
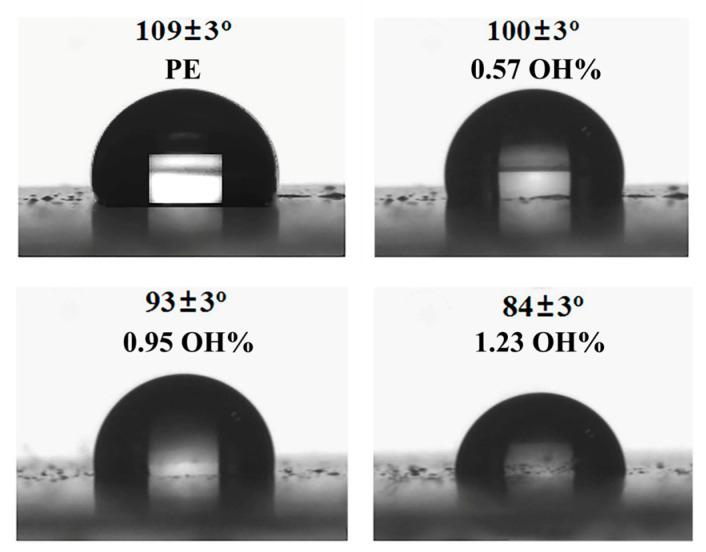
The water contact angle of hydroxyl-functionalized polyethylenes (entries 5–7 in Table 1).

The introduction of a small amount of polar groups in polyolefins can dramatically improve material properties such as compatibility, paintability, printability, and adhesion [79,80]. The surface properties of hydroxyl-functionalized polyethylenes (E/U-OH copolymers) with different -OH incorporations were evaluated by measuring the water contact angle (WCA). As shown in Figure 7, the WCA of pure branched polyethylene is 110° [62,68], while the WCA of copolymer decreased dramatically with the increasing hydroxyl incorporation. The lowest WCA (84°) was obtained when the hydroxyl incorporation was 1.23 mol% [81]. Therefore, the surface of the PE material can be changed from hydrophobic to hydrophilic with the introduction of a small amount of U-OH.

## 3. Materials and Methods

### 3.1. General Procedures

All manipulations involving air- and moisture-sensitive compounds were carried out under an atmosphere of dried and purified nitrogen using standard vacuum-line, Schlenk, or glovebox techniques.

### 3.2. Materials

Toluene was refluxed over Na/K alloy before use, and dichloromethane was dried over P_2_O_5_ and was distilled under nitrogen. Et_2_AlCl (0.9 M in Toluene) was purchased from Acros. Ethylene (99.99%) was purified by passing through Agilent moisture and oxygen traps. Methyl 10-undecenoate (U-COOMe), 10-undecen-1-ol (U-OH), and 10-undecenyl bromide (U-Br) were purchased from Aldrich. Other commercially available reagents were purchased and used without purification.

The dibenzobarrelene *α*-diimine nickel complex, **Ni-DBB,** was prepared according to our previous work [47]. The *α*-diimine ligand **DBB-L** was first synthesized and fully confirmed by ^1^H and ^13^C NMR. ^1^H NMR (CDCl_3_, 400 MHz), δ (ppm): 7.25–7.02 (m, 14H, Ar-***H***), 4.98 (s, 2H, C***H***), 2.49 (m, 4H, C***H***(CH_3_)_2_), 1.15 (d, 12H, C***H***_3_), 1.02 (d, 12H, C***H***_3_). ^13^C NMR (CDCl_3_, 100 MHz), δ (ppm): 158.46, 145.56, 138.54, 136.36, 127.29, 125.38, 124.14, 122.81, 51.12, 23.29, 22.48. Nickel complex **DBB-Ni** was obtained by the addition of the ligand **DBB-L** to a stirring suspension of (dimethoxyethane)NiBr_2_ in CH_2_Cl_2_ and confirmed by elemental analysis. Anal. Calcd for C_40_H_44_Br_2_N_2_Ni: C, 62.29; H, 5.75; N, 3.63; Found: C, 62.36; H, 5.66; N, 3.51.

### 3.3. Measurements

The NMR analysis of the copolymers was performed on a Bruker Avance III HD 400 MHz spectrometer. The incorporation (mol%) of U-COOMe in the copolymer was calculated by integrating methyl proton signals (-OC***H***_3_) of inserted U-COOMe with respect to signals of other protons in the ^1^H NMR spectrum (incorporation mol% = 4*I*_OC***H***3_/3(*I*_C***H***3_ + *I*_C***H***2_ + *I*_C***H***_) × 100%) [48]. The incorporation (mol%) of U-OH and U-Br in the copolymer was calculated by integrating methylene proton signals (-C***H***_2_-) of inserted U-OH/U-Br with respect to signals of other protons in the ^1^H NMR spectrum (incorporation mol% = 2I_C***H***2_/(*I*_C***H***3_ + *I*_C***H***2_ + *I*_C***H***_) × 100%) [66,67]. The branching density per 1000 carbon atoms of copolymers was determined by ^1^H NMR spectrum. The branching distribution was quantitatively calculated based on previously reported resonance assignments [76,77]. For each distinguishable type of branch in the ¹³C NMR spectrum, a representative signal was selected, and the content of each branch type was determined by integrating the area under the corresponding peak. Differential scanning calorimeter (DSC) analyses of PEs were conducted with a PerkinElmer DSC-4000 system. The DSC curves were recorded as second heating curves from −100 °C to +150 °C at a heating rate of 10 °C/min and a cooling rate of 10 °C/min. Recorded at second heating curves to examine the thermal behavior of the copolymers. GPC analyses of the molecular weights and molecular weight distributions (PDI = *M*_w_/*M*_n_) of the PE samples at 150 °C were performed on a PL-GPC 220 high-temperature chromatograph equipped with two GPC columns (PLgel 10 μm MIXED-B, Santa Clara, CA, USA), (Agilent Technologies, Palo Alto, CA, USA) and a differential refractive-index detector. 1,2,4-Trichlorobenzene (TCB) (Agilent Technologies, Palo Alto, CA, USA) was used as the eluent at a flow rate of 1.0 mL/min. The molecular weight data were analyzed using narrow polystyrene standards (Polymer Laboratories, Long Beach, CA, USA) and were corre cted for polymer samples by universal calibration. The water contact angle (WCA) test was conducted on a goniometer (DSA100S) (KRÜSS Scientific, Hamburg, Germany). Elemental analyses were performed on a Vario EL microanalyzer.

### 3.4. Procedure for the Copolymerization of Ethylene and Comonomers

A round-bottom Schlenk flask with stirring bar was heated for 2 h at 150 °C under vacuum and then cooled to room temperature. The flask was pressurized to 1.2 atm of ethylene and vented three times. Then the glass reactor was charged with the required amount of freshly distilled toluene, Et_2_AlCl, and polar monomer in sequence under 1.2 atm of ethylene. The system was continuously stirred for 5 min at the desired temperature, and then 1 mL of a solution of nickel complex in CH_2_Cl_2_ was added. The total reaction volume was kept at 21 mL. The ethylene pressure was kept constant at 1.2 atm. Polymerization was terminated by the addition of acidic methanol after releasing ethylene pressure. The resulting precipitated polymers were collected and treated by filtering and washed with methanol several times. The final product was dried under vacuum at 40 °C to a constant weight.

## 4. Conclusions

In summary, an *α*-diimine nickel catalyst with a bulky dibenzobarrelene backbone was able to catalyze the copolymerization of ethylene and biosourced comonomers derived from castor oil, such as methyl 10-undecenoate (U-COOMe), 10-undecen-1-ol (U-OH), or 10-undecenyl bromide (U-Br). Copolymerization activity increased, but the incorporation of polar comonomers decreased in the order of U-COOMe > U-OH > U-Br. Besides, the copolymer molecular weight followed the order of U-OH > U-COOMe > U-Br. The copolymerization activity and the molecular weight of copolymers decreased, but the incorporation of monomer increased with an increase in temperature. Increasing the comonomer concentration led to an increase in the incorporation of comonomers, whereas the copolymerization activity and the molecular weight of copolymers decreased. Controlled copolymerization of ethylene and U-OH was realized at 20 °C to afford a narrowly dispersed copolymer. The produced functional polyethylenes are highly branched, and their hydrophilic properties improve with increasing incorporation of polar groups. This study provides access to synthesize bio-based functional polyolefins by copolymerization of ethylene and biosourced comonomers using earth-abundant nickel catalysts.

## Figures and Tables

**Table 1:** Copolymerization results of ethylene and comonomers under various temperatures *^a^*.

Entry	Monomer	T (°C)	Yield (mg)	Act. *^b^*	Incorporation* ^c^* (mol%)	*M*_n_ *^d^* (kg/mol)	PDI *^d^*	BD *^e^*
1	U-COOMe	20	150	150	0.78	104.2	1.15	86
2	U-COOMe	35	122	122	1.01	81.4	1.18	89
3	U-COOMe	50	85	85	1.42	42.3	1.40	91
4	U-COOMe	65	trace	-	-	-	-	-
5	U-OH	20	340	340	0.57	121.7	1.07	88
6	U-OH	35	231	231	0.95	105.6	1.20	87
7	U-OH	50	213	213	1.23	70.2	1.35	82
8	U-OH	65	trace	-	-	-	-	-
9	U-Br	20	360	360	0.33	59.4	1.51	81
10	U-Br	35	303	303	0.36	36.8	1.53	85
11	U-Br	50	260	260	0.41	18.4	1.54	87
12	U-Br	65	218	218	0.47	9.1	1.65	95
13	U-COOH	20	trace	-	-	-	-	-

*^a^* Polymerization conditions: 2 μmol of Ni, Al(AlEt_2_Cl)/Ni =1600, monomers (mol/L) = 0.1, 1.2 atm, 30 min, 20 mL toluene, and 1 mL CH_2_Cl_2_. *^b^* Activity in kg copolymer/(mol Ni·h). *^c^* Determined by ^1^H NMR spectroscopy. *^d^* Determined by GPC in 1,2,4-trichlorobenzene at 150 °C. *^e^* Branching density (/1000 C), determined by ^1^H NMR spectroscopy.

**Table 2:** Copolymerization results of ethylene and comonomers with various concentrations *^a^*.

Entry	Monomer	Conc. (mol/L)	Yield (mg)	Act. *^b^*	Incorporation* ^c^* (mol%)	*M*_n_ *^d^* (kg/mol)	PDI *^d^*	BD *^e^*
1	U-COOMe	0.05	170	170	0.42	125.2	1.12	88
2	U-COOMe	0.1	150	150	0.78	104.2	1.15	86
3	U-COOMe	0.2	106	106	1.31	89.1	1.24	83
4	U-OH	0.05	474	474	0.34	145.2	1.05	89
5	U-OH	0.1	340	340	0.57	121.7	1.07	88
6	U-OH	0.2	102	102	1.06	54.1	1.86	78
7	U-Br	0.1	360	360	0.33	59.4	1.51	81
8	U-Br	0.2	280	280	0.62	30.0	1.55	81
9	U-Br	0.3	209	209	0.93	23.7	1.61	80

*^a^* Polymerization conditions: 2 μmol of Ni, Al(AlEt_2_Cl)/Ni =1600, 20 °C, 1.2 atm, 30 min, 20 mL toluene and 1 mL CH_2_Cl_2_. *^b^* Activity in kg copolymer/(mol Ni·h). *^c^* Determined by ^1^H NMR spectroscopy. *^d^* Determined by GPC in 1,2,4-trichlorobenzene at 150 °C. *^e^* Branching density (/1000 C), determined by ^1^H NMR spectroscopy.

## Data Availability

The data presented in this study are available on request from the corresponding author.
